# Prevalence of and Associated Risk Factors for High Risk Human Papillomavirus among Sexually Active Women, Swaziland

**DOI:** 10.1371/journal.pone.0170189

**Published:** 2017-01-23

**Authors:** Themba G. Ginindza, Xolisile Dlamini, Maribel Almonte, Rolando Herrero, Pauline E. Jolly, Joyce M. Tsoka-Gwegweni, Elisabete Weiderpass, Nathalie Broutet, Benn Sartorius

**Affiliations:** 1 Department of Public Health, School of Nursing and Public Health, University of KwaZulu-Natal, Durban, South Africa; 2 Epidemiology Unit, Ministry of Health, Mbabane, Swaziland; 3 International Agency for Research on Cancer (IARC), Prevention and Implementation Group, Lyon, France; 4 Department of Epidemiology, University of Alabama at Birmingham, Birmingham, Alabama, United States of America; 5 Department of Research, Cancer Registry of Norway, Institute of Population-Based Cancer Research, Oslo, Norway; 6 Department of Community Medicine, Faculty of Health Sciences, University of Tromsø, The Arctic University of Norway, Tromsø, Norway; 7 Department of Medical Epidemiology and Biostatistics, Karolinska Institutet, Stockholm, Sweden; 8 Genetic Epidemiology Group, Folkhälsan Research Center, Helsinki, Finland; 9 World Health Organization; Department of Reproductive Health and Research, Geneva, Switzerland; Bharathidasan University, INDIA

## Abstract

**Background:**

High risk human papillomavirus (hr-HPV) infection and the dual burden of HIV remains a huge challenge in some low-income countries (LICs) such as Swaziland with limited or no data. We estimated the prevalence and investigated determinants of hr-HPV, including HIV infection among sexually active women in Swaziland.

**Methods:**

A total of 655 women aged between 15 and 49 years from five health facilities were randomly enrolled using a cross-sectional study design. Cervical cells were tested for hr-HPV types using GeneXpert HPV Assays.

**Results:**

The overall weighted hr-HPV prevalence was 46.2% (95%CI: 42.8–49.5). Of hr-HPV infected women, 12.4% (95%CI: 8.6–17.5) were HPV16-positive, 13.8% (95%CI:12.0–15.8) were positive for HPV18/45, 26.7% (95%CI: 24.2–29.3) for HPV31/33/35/52/58, 7.6% (95%CI: 7.6–11.9) for HPV51/59 and 11.0%, (95%CI: 7.9–15.3) for HPV39/56/66/68. Prevalence of hr-HPV decreased with increasing age. Overall HIV prevalence remained high (42.7%; 95%CI: 35.7–46.2). HIV infection was associated with hr-HPV infection (Adjusted OR = 4.9, 95%CI: 3.043–7.8, p<0.001). Overall hr-HPV/HIV co-infection was 24.4% (95%CI: 20.3–29.1) which was significantly higher among younger age groups (p<0.001). Prevalence of multiple group hr-HPV infection was significantly higher in HIV-positive versus -negative women (27.7% and 12.7% respectively, p<0.001). The presence, absence or unknown of history of STI with HIV did not appear to modify the relationship with hr-HPV (OR = 4.2, 95%CI: 2.6–7.1, OR = 4.6, 95%CI: 2.8–7.7, p<0.001, p<0.001 and OR = 4.1, 95%CI: 1.3–13.4, p<0.021 respectively).

**Conclusion:**

The prevalence of hr-HPV infection was high and significantly associated with HIV among sexually active women. Furthermore, the study has provided essential information about the HIV link with hr-HPV infections which may explain the high prevalence among HIV infected women. This can contribute to policy development and planning of prevention strategies incorporating HPV infection prevention especially among youth and HIV infected people.

## Introduction

In low-income countries (LICs), the burden of cervical cancer is a serious health problem and predominantly affects women of reproductive age [1]. Cervical cancer (CC) is the fourth most common cancer, and about (87%) of cases occurred in LICs in 2012 [2]. Out of the 40 Human Papillomavirus (HPV) genotypes that infect the genital tract, 13 are considered to be "high-risk" and approximately 80% of women worldwide become infected with at least one type before reaching 50 years [3]. High risk HPV (hr-HPV) is the major and necessary cause of CC [4]. HPV16 and 18 are associated with approximately 71% of all cases of CC, and HPV45 is associated with approximately 6% of additional cases of CC [5]. The prevalence of HPV infection in women varies greatly in the African continent where some of the highest prevalences are found [6]. Both hr-HPV and HIV are sexually transmitted infectious agents, and infection by one of the viruses may accelerate transmission of the other [7]. The impact of co-infection of HPV with HIV in the Sub-Saharan African countries has created a huge burden of cervical abnormalities since HIV infected women have higher prevalence of hr-HPV [8]. The latter is more likely to be persistent in HIV-infected women, and results in a higher incidence of high-grade squamous intraepithelial lesion (HSIL) [9].

The high prevalence of HPV infection among women in several African countries may be explained by the presence of risk factors such as early age sexual debut, the number of sexual partners of women and of their partners, and other STIs, including HIV, as discussed above [10–13]. Previous studies have shown that the prevalence of HPV infection rises soon after the onset of sexual activity [14, 15] and peaks in adolescence and early adulthood after which it declines during later decades of life [16]. The high prevalence of HPV infection among young women has been found to be due to the absence of adaptive immune response and the relatively larger area of cervical epithelium undergoing squamous metaplasia [17].

Studies estimating HPV infection are limited in African countries [4]. The lack of hr-HPV identification for CC screening has delayed the improving and expansion of triage, treatment and prevention in LICs like Swaziland. Information on country-specific HPV genotype prevalence is needed to inform local policy, screening and prevention programmes. Randomised trials have demonstrated that HPV-based screening is more effective than cytological screening in reducing the incidence of invasive squamous and adeno-carcinoma of the cervix uteri [18, 19]. The current prevalence of HPV infection in Swaziland remains unknown. Epidemiological data on prevalence, distribution of HPV types and HPV-related conditions are vitally important to reduce the burden of CC by (1) guiding the introduction of prophylactic vaccines, (2) strengthening the screening programme, (3) promoting the development of cancer registration, and (4) initiating a national cancer prevention and control policy discussion.

Therefore, the main aim of this study was to estimate prevalence and identify associated determinants of hr-HPV, including HIV infection among reproductive aged women in Swaziland.

## Materials and Methods

### Study setting and population

**Fig 1:**
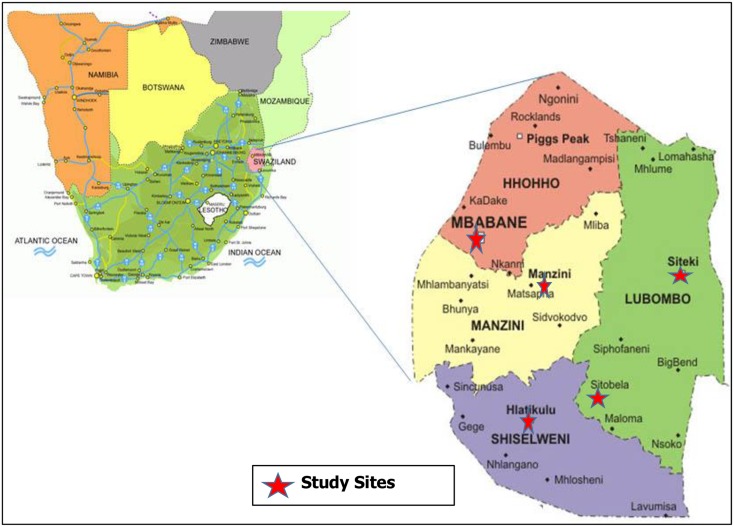
Map showing the location, political regions of Swaziland and health facilities (study sites with a red star). **Source:** Macmillan. Geography and map of Swaziland Matsapha2005 [accessed 2012 13]. Available from: http://geography.about.com/library/blcswaziland.htm.

The study participants were women aged between 15 and 49 years, attending healthcare facilities for routine health care-related services in June–July 2015. All women with a history of previous or current sexual activity who provided written informed consent were included. The women were recruited from five healthcare facilities (Mbabane Government, Realign Fitkin Memorial (RFM), Hlatsikhulu, Sithobela and Siteki Public health unit) within the four political regions of Swaziland that had fully functioning CC screening services such as VIA and cryotherapy (Fig 1).

### Sample size

To estimated prevalence of the outcomes of interest, assuming 95% confidence and an acceptable margin of error of 5%, and maximum variability i.e. 50% (given unknown prevalence) a sample size of 384 subjects was required were used. The sample was further increased by a margin of 10% to account for potential non-response and multiplied by a design effect (D) of 1.5. The final sample size of the study was 650 women.

### Sampling strategy

The recruitment of participants was done in two sampling stages; firstly women were stratified by age (seven age-groups) and then sampled using systematic random sampling (every 3^rd^ woman of those first selected) using the lottery method. The participants were selected from each site until the calculated sample size was achieved per site.

### Data collection

A structured standardized questionnaire was administered by a trained nurse, prior to clinical examination and specimen collection, to obtain detailed data on socio-demographic characteristics, sexual, reproductive and gynaecological histories. Prior to clinical examination and specimen collection, the nurse midwife inspected perineal, vulvar, vaginal and cervical regions of each woman for evidence of warts, ulcers, discharge, inflammation or tenderness, and recorded all abnormalities according to the study protocol. Data were entered and stored into EpiData 3.02 for Windows (The Epi Data Association Odense, Denmark). Each participant was assigned a unique study identity number that was used to link the questionnaire with the specimens. Personal information blinded for researchers was kept on site for results feedback and validation of data.

### Specimen collection and Testing

After visual inspection of the vulva, a non-lubricated sterile disposable speculum was inserted and cervical cells were collected using a sterile cyto-brush by rotating the brush 3-times 360° in the cervix (Copan International, Brescia, Italy). The head of the cervical brush was then eluted in a labeled vial containing Thin Prep Preservcyt Preservation solution to assess the presence of hr-HPV and for the Liquid-Based cytology (ThinPrep;Hologic-Cytyc Company, Marlborough, Mass). After HIV pre-counselling, 4ml of blood were collected in a vacutainer tube from participants consenting to HIV testing. All specimens were collected and transported daily to the national laboratory. All tests were performed at the Swaziland Government National Referral Laboratory, Mbabane.

### HPV testing

The HPV-DNA testing was done using the GeneXpert HPV assay (Xpert HPV Assay) (Cepheid, Sunnyvale, CA, 2014) according to the manufacturer’s protocol. The Xpert HPV Assay is a qualitative *in vitro* test for the detection of the E6/E7 region of the viral DNA genome from hr-HPV in patient specimens. The Xpert HPV test gives results from 6 separate channels: (i) sample adequacy control (SAC), (ii) P1-HPV16, (iii) P2-HPV18/45, (iv) P3-HPV 31/33/35/52/58, (v) P4-HPV51/59 and (vi) P5-HPV39/68/56/66. An individual specimen can be positive for more than one probe. For the purpose of our analysis infection, positivity on one probe only was classified as single group hr-HPV infection (though with the possibility of multiple HPV type infection with P2-P5). Multiple group hr-HPV infection was classified as positivity on two or more probes.

### HIV Testing

The Alere Determine HIV-1/2 Ag/Ab Combo test was used to detect both HIV-1/2 antibodies and free HIV-1 p24 antigen. Reactive specimens were confirmed by Trinity Biotech Uni-Gold Recombigen HIV Test. All participants who tested HIV-positive were post-counselled and referred to health units offering the necessary HIV treatment, care and support services.

### Data analysis

Data were processed and analysed using Stata 13.0SE (Stata corp.College station, Texas, USA). Data were checked for possible errors and missing values prior to analysis. Age and region-weighted analyses were done to estimate the overall hr-HPV prevalence and co-infection with HIV infection given the stratified systematic random sampling design. Survey weighted Analysis was done to adjust the sample characteristic to match up with the target population (15–49) that they were selected to represent. It’s applied to bring the proportion of women in the sample in the alignment with the portion of women in the target population. Therefore all results reported as weighted. Survey weighted prevalence and 95% confidence interval (CI) were calculated. In addition, assuming that our study subjects are representative of the population of Swaziland, we used survey weights to extrapolate sample proportions to population totals (age-region weighted) to estimate burden counts based on the 2007–2030 population projections aligned to the 2014 population estimates total of 377169 women aged 15–49 years [20]. Differences in prevalence by categorical variables such as site, age, and HIV were assessed using the survey weighted chi-squared (χ^2^) test. Odds ratios (unadjusted and adjusted) and 95%CIs for potential risk factors associated with hr-HPV were estimated using logistic regression model. Variables significant at significant cut-off of 0.2 in the bivariate regression analyses were selected for inclusion into the final multivariable model. An adjusted p-value of <0.05 was deemed statistically significant.

### Ethical consideration

The study was approved by the Swaziland Scientific Ethics Committee (MH599C/FW00015267/IRB0009688) and the Biomedical Research Ethics Committee of the University of KwaZulu-Natal (BE 242/14). Ethics committees approved written informed consent which was obtained from all the participants prior participating to the study. All sexually active women aged between 15 and 49 years attending various reproductive health clinics and other units from all the study sites, were eligible for the study. In Swaziland according to national guidelines for HIV testing and counselling, the consenting age is 12 years [21].

## Results

### Characteristics of the study population

A total of 655 women were enrolled in the study in June–July 2015. A total of 644 subjects produced sufficient specimen for hr-HPV testing i.e. 11 were thus excluded from further analyses. The mean (± standard deviation [SD]) age for enrolled women was 32.2 (±8.7) years, and at menarche 14.4 (±1.7) years, at first intercourse 17.9 (±2.9) years, and at first pregnancy 19.4 (±3.9) years. Of the 644 participants, 571 (88.7%) had been pregnant previously, 542 (84.2%) had a history of contraceptive use, 272 (42.6%) had a history of STIs, 116 (18.0%) had STIs that had been treated in the past 12 months, 345 (53.6%) were married or cohabiting, 373(57.9%) had attended secondary/high school, 340(53.0%) were unemployed and 513(79.7%) reported one lifetime sexual partner (Table 1).

**Table 1:** Socio-demographic characteristics of the study population (N = 644).

Demographics	n (%)
**Age: n (%)**	
Mean ± SD (range)	**32 .2 ± 8.7**
15–19	39 (6.1)
20–24	111 (17.2)
25–29	131 (20.3)
30–34	116 (18.0)
35–39	103 (16.0)
40–44	80 (12.4)
45–49	75 (11.6)
**Marital status: n (%)**	
Single	266 (41.3)
Cohabiting	38 (5.9)
Married	307 (47.7)
Divorced/separated	22 (3.4)
Widow	11 (1.7)
**Education: n (%)**	
Never been to school	24 (3.7)
Primary	130 (20.2)
Secondary/High	373 (57.9)
Tertiary	117 (18.2)
**Occupation: n (%)**	
Unemployed	340 (53.0)
Employed	255 (39.8)
Self-employed	46(7.2)
**Pregnant before: n (%)**	
Yes	571 (88.7)
No	64 (9.9)
Missing	9 (1.4)
**Age at first pregnancy: Mean age (SD)**	19.4 (3.9)
**No. pregnancies: n (%)**	
0	6 (1.0)
1	139 (24.3)
2	127 (22.2)
3+	297(46.1)
**Age Menarche**: Mean (SD)	14.39 (1.7)
**Age at first intercourse**: Mean age (SD)	17.90 (2.9)
**Number of Sexual life partner: n (%)**	
0	33 (5.1)
1	513 (79.7)
2	61 (9.5)
3+	43 (6.7)
**Contraceptives use: n (%)**	
Yes	542 (84.2)
No	95 (14.8)
Missing	7 (1.1)
**Ever had STI: n (%)**	
Yes	272 (42.6)
No	337 (52.7)
Don’t remember	30 (4.7)
**STIs treated in the past 12 months: n (%)**	
Yes	116 (18.0)
No	520 (80.9)
Don’t know	7 (1.1)
**HIV Status: n (%)**	
Positive	276 (42.7)
Negative	368 (57.1)

### Hr-HPV prevalence

**Fig 2:**
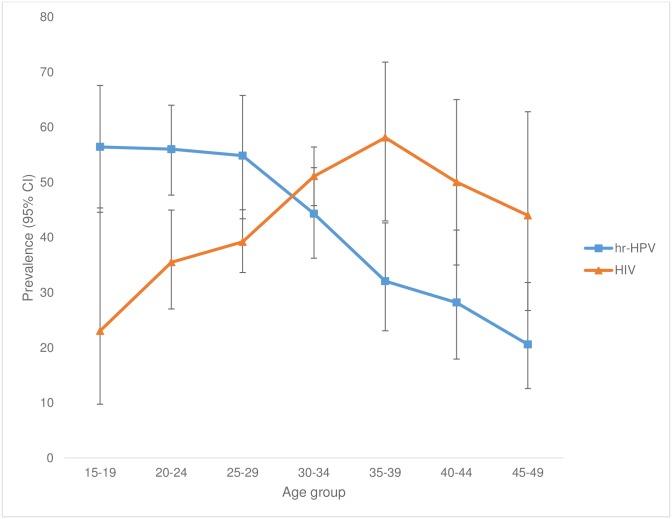
Age-specific prevalence of HPV and HIV among reproductive age women in Swaziland.

The overall weighted hr-HPV prevalence was 46.2% (95%CI: 42.8–49.5) (Table 2). Of hr-HPV infected women, 12.4% (95%CI: 8.6–17.5) were HPV16-positive, 13.8% (95%CI: 12.0–15.8) were positive for HPV18/45, 26.7% (95%CI: 24.2–29.3) for HPV31/33/35/52/58, 7.6% (95%CI:7.6–11.9) for HPV51/59 and 11.0%, (95%CI: 7.9–15.3) for HPV39/56/66/68 (Table 2). The highest peaks of hr-HPV infection were noticed among women younger than 30 years (55.4% in 15**–**19 years old, 54.5% in 20**–**24 years old and 55.8% in 25**–**29 years old) as compared to older women (aged between 30 and 49 years). The prevalence declined with increasing age (Table 3 and Fig 2). The highest hr-HPV prevalence was detected among women from Siteki PHU (52.8%, 95%CI: 39.5–65.7), although the difference across the sites was not significant (p = 0.84) (Table 3).

**Table 2:** The weighted prevalence and estimated population burden of hr-HPV infection among sexual reproductive women (15–49 years) in Swaziland (n = 644).

Hr-HPV types	Positive (N = 644)^i^	Crude prevalence(%)	Survey weighted prevalence(%, 95%CI)	Population burden^j^	95%CI
**All hr-HPV types**	273	42.1	46.2 (42.79–49.53)	174046	153294–194797
HPV16	64	9.7	12.4 (8.61–17.45)	46642	28097–65188
HPV18/45	78	12.0	13.8 (11.95–15.83)	51959	45976–57942
HPV31/33/35/52/ 58	157	24.3	26.7 (24.23–29.27)	100598	86384–114811
HPV51/59	43	6.6	7.6 (4.9–11.91)	28730	16672–40787
HPV39/56/66/68	67	10.3	11.0 (7.86–15.29)	41630	29163–54097
**Single group hr-HPV infection**^a^	170	26.4	27.4 (24.72–30.21	103260	86550–119970
**Multiple group hr-HPV infection**^b^	103	16.0	18.8 (15.71–22.27)	70786	58763–82808

^i^: N = 655 but eleven samples were not valid for HPV testing

^j^: Population burden estimates to extrapolate absolute burden counts were made based on the 2007**–**2030 population projections aligned to the 2014 population estimates

^a^: Single group hr-HPV positivity on one probe only was classified as single group hr-HPV infection (though with the possibility of multiple HPV type infection with P2-P5).

^b^: Multiple group hr-HPV infection: classified as positivity on two or more probes.

NB: The totals of HPV types positive do not add up to the total of all hr-HPV types because some have multiples types as grouped by the GeneXpert HPV assay

**Table 3:** Overall hr-HPV, HIV and hr-HPV/HIV co-infection prevalence by age groups and study site (Crude and weighted) (N = 644).

Characteristic	*Crude hr-HPV+ve (n, %) (N = 644)	hr-HPV+ve (weighted) (%, 95%CI)	*Crude HIV+ve (n, %)(N = 644)	HIV+ve^j^ (weighted) (%, 95%CI)	hr-HPV and HIV co-infection		
					hr-HPV+ve only	HIV+ve only	hr-HPV & HIV +ve
**Age group**							
**15–19**	25 (56.8)	55.4 (44.5–65.7)	10 (22.7)	24.5 (12.3–42.8)	37.7 (29.4–46.8)	6.8 (2.7–15.9)	17.7 (7.5–36.4)
**20–24**	60 (55.6)	54.5 (46.8–61.9)	35 (32.4)	35.2 (26.8–44.7)	26.0 (18.1–35.9)	6.8 (2.8–15.5)	28.5 (19.9–38.9)
**25–29**	66 (54.1)	55.8 (44.0–66.9)	48 (39.3)	39.0 (34.1–44.2)	27.3 (18.6–38.2)	10.9 (5.2–21.6)	28.5 (23.7–33.9)
**30–34**	62 (43.7)	44.2 (34.7–54.1)	72 (50.7)	51.4 (44.2–58.6)	14.0 (7.1–26.1)	21.2 (14.6–29.9)	30.2 (21.8–40.4)
**35–39**	28 (29.8)	31.1 (22.8–40.8)	51 (54.3)	58.1 (40.7–73.7)	6.5 (2.4–16.5)	33.6 (25.9–42.2)	24.6 (15.8–36.0)
**40–44**	20 (26.3)	30.0 (21.8–39.7)	36 (47.4)	51.3 (36.6–65.8)	7.5 (3.2–16.6)	27.0 (19.8–35.5)	22.5 (11.4–39.8)
**45–49**	12 (20.7)	20.59 (42.5–49.4)	24 (41.4)	44.0 (27.3–62.2)	6.2 (2.7–13.9)	29.6 (13.6–53.0)	14.4 (7.7–25.2)
**Study Sites**							
**Mbabane (H01)**	78 (43.8)	45.5 (32.4–59.2)	68 (38.2)	33.2 (22.4–46.0)	25.3 (17.3–35.3)	13.4 (04.8–32.0)	19.6 (16.4–23.3)
**RFM (H02)**	76 (39.6)	40.7 (35.2–46.5)	96 (50.0)	44.3 (27.4–62.6)	15.3 (0.7–31.9)	19.2 (11.9–29.5)	25.0 (15.8–37.3)
**Siteki PHU (H03)**	34 (50)	52.8 (39.5–65.7)	31 (45.6)	41.3 (21.8–64.0)	28.0 (13.3–49.7)	16.5 (0.9–28.8)	24.8 (13.0–42.1)
**Sithobela (H04)**	30 (44.8)	49.1 (32.1–66.4)	32 (47.8)	45.3 (31.4–60.0)	20.0 (0.9–38.3)	16.1 (0.5–40.9)	29.1 (18.9–42.1)
**Hlathikhulu (H05)**	55 (39.6)	47.4 (31.1 –- 64.0)	49 (35.3)	39.0 (30.9–47.8)	22.3 (12.1–37.4)	13.9 (0.8–23.7)	25.1 (20.4–30.5)
**Overall**	273 (42.4)	**46.2 (42.5–49.4)**	276 (42.9)	**42.7 (35.7–46.2)**	**21.5 (17.7–25.9)**	**16.1 (12.9–19.9)**	**24.4 (20.5–28.7)**

^i^HIV+ve: HIV-positive

^j^hr-HPV+ve: hr-HPV-positive

*crude: The prevalence based on the study sample size (N = 644).

### Population burden of hr-HPV

Based on sampling weights, the overall population burden of hr-HPV among women aged between 15 and 49 years was estimated at 174 046 (95%CI: 153 294–194 797). When stratified by types 46 642 (95%CI: 28 097–65 188) were estimated to have HPV16, 51 959 (95%CI: 45976–57942) HPV18/45, 100 598 (95%CI: 86 384–114 811) HPV31/33/35/52/58, 28 730 (95%CI: 16 672–40 787) HPV51/59 and 41 630 (95%CI: 29 163–54 097) HPV39/56/66/68 (Table 2). Multiple group hr-HPV infection were detected in 18.8% (95%CI: 15.71–22.27) of subjects while 27.4% (95%CI: 24.72–30.21) were infected with a single group hr-HPV infection (Table 2). Population burden of single group hr-HPV was estimated at 103 260 women aged 15–49 (95%CI: 86 550–119 970) and 70786 (95%CI: 58 763–82 808) with multiple group hr-HPV infection (Table 2).

### HIV prevalence and Hr-HPV/HIV co-infection

**Fig 3:**
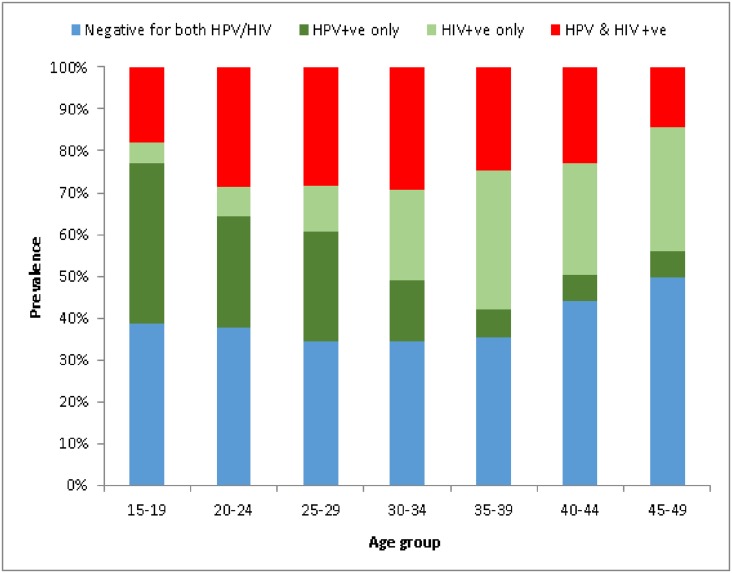
The trend of hr-HPV types and HIV infection by age-group.

The overall HIV weighted prevalence was 42.7% (95%CI: 35.7–46.2). The HIV prevalence increased with age; from 24.5% (95%CI: 12.3–42.8) in the 15–19 year age group, to a peak of 58.1% (95%CI: 40.7–73.7) in the 30–34 year age group, and then declined in the 45–49 year age group (44.0%; 95%CI: 27.3–62.2) (Table 3). The overall hr-HPV/HIV co-infection was 24.4% (95%CI: 20.5–28.7) and it was significantly higher among the young age groups than the older age groups (p<0.001) (Table 3). While hr-HPV prevalence significantly decreased with age, HIV prevalence significantly increased with age (p<0.001) (Fig 2). The age-specific prevalence for hr-HPV/HIV co-infection was significantly high among the 20–30 age group with the highest peak among the 30–34 age group and declined in older age groups (p<0.001) (Fig 3). Age-specific prevalence of Hr-HPV among reproductive aged women who were HIV-positive was significantly higher as compared to HIV-negative women (78% in the 15–19 age, 80% in the 20–24 age, 72% in the 25–29 age and 58% in the 30–34 age) (S1 Fig). Also, the prevalence of multiple group hr-HPV types was significantly higher in HIV-positive women (27.7%, 95%CI: 22.6–33.6) compared to HIV-negative women (12.7%, 95%CI: 15.7–22.3) (p<0.001) (data not shown). HPV16 and HPV31/33/35/52/58 were significantly higher among HIV-positive women as compared to HIV-negative women (17.6%, OR = 2.2, 95%CI: 1.1–4.5, p = 0.029, and 40.4%, OR = 3.2, 95% CI: 1.9–5.5, p <0.001, respectively) (S1 Table).

### Risk factors associated with hr-HPV infection

Table 4 shows risk factors associated with hr-HPV infection. Based on the univariate analysis: increasing age (OR = 0.94, 95%CI: 0.93–0.96, p<0.001), being married (OR = 0.6, 95%CI: 0.4–0.8, p = 0.003) and increasing number of pregnancies (OR = 0.9, 95%CI: 0.8–0.9, p = 0.001) were inversely associated with hr-HPV. Only HIV-positive status was positively associated with hr-HPV (OR = 2.9, 95%CI: 2.1–4.0), p<0.001). Even after multivariable adjustment, the risk of hr-HPV decreased with increasing age (OR = 0.9, 95%CI: 0.92–0.94, p<0.001). Those living with partners had a decreased risk of being hr-HPV infected (OR = 0.4, 95%CI, 0.2–0.9, p = 0.027), and being HIV-positive remained a highly prominent risk factor for hr-HPV infection (OR = 4.1, 95%CI: 2.8–6.0, p<0.001). The presence, absence or unknown of history of STI with HIV did not appear to modify the relationship with hr-HPV (OR = 4.2, 95%CI: 2.6–7.1, OR = 4.6, 95%CI: 2.8–7.7, p<0.001, p<0.001 and OR = 4.1, 95%CI: 1.3–13.4, p<0.021 respectively).

Education status, parity, age at first sexual encounter and history of STIs treated in the past 12 months were not associated with hr-HPV after multivariable adjustment.

**Table 4:** Risk factors associated with hr-HPV infection (N = 644).

Risk factors	hr-HPV+ve		Unadjusted (univariate)		Adjusted (multivariable)i	
	n/N	(%)	OR (95%CI)	P-value	OR (95%CI)	P-value
**Age**						
Mean ± SD (range)	273/644	42.4	**0.94 (0.93–0.96)**	**<0.001**	**0.92 (0.90–0.94)**	**<0.001**
**Marital status**						
Single	132/266	49.6	1 (ref)		1 (ref)	
Living with partner/Cohabiting	13/38	34.2	0.5 (0.3–1.1)	0.079	**0.4 (0.2–0.9)**	**0.027**
Married	115/307	37.5	0.6 (0.4–0.8)	**0.003**	1.0 (0.7–1.5)	0.982
Divorced/separated	9/22	40.9	0.7 (0.3–1.7)	0.434	1.3 (0.5–3.7)	0.562
Widow	4/11	36.4	0.6 (0.2–2)	0.394	1.5 (0.4–5.9)	0.607
**Education**						
Never been to school	10/24	41.7	1 (ref)			
Primary	60/130	46.2	1.2 (0.5–2.9)	0.685		
Secondary/High	153/373	41.0	1 (0.4–2.2)	0.95		
Tertiary	50/117	42.7	1 (0.4–2.5)	0.923		
**Occupation**						
Unemployed	153/340	45.0	1 (ref)		1 (ref)	
Employed	104/255	40.8	0.8 (0.6–1.2)	0.304	1.2 (0.82–1.75)	0.355
Self-employed	15/46	32.6	0.6 (0.3–1.1)	0.115	0.64 (0.3–1.35)	0.239
**Pregnant before**						
Yes	239/571	41.9	1 (ref)			
No	31/64	48.4	1.3 (0.8–2.2)	0.314		
**No. of pregnancies (Mean)**	273/644	42.4	0.9 (0.8–0.9)*	0.001*		
**Age Menarche (Mean age)**	273/644	42.4	1.1 (1–1.2)	0.13		
**Age at first sex (mean age)**	273/644	42.4	1 (1–1.1)	0.654		
**Sexual life partner**						
0	11/33	33.3	1 (ref)		1 (ref)	
1	214/513	41.7	1.4 (0.7–3)	0.345	0.94 (0.4–2.21)	0.883
2	31/61	50.8	2.1 (0.9–5)	0.106	1.04 (0.38–2.85)	0.938
3+	17/36	47.2	1.8 (0.7–4.7)	0.242	0.95 (0.31–2.88)	0.924
**Contraceptives use**						
Yes	225/542	41.5	1 (ref)			
No	44/95	46.3	1.2 (0.8–1.9)	0.382		
**Ever had STI**						
Yes	113/272	41.5	1 (ref)			
No	142/337	42.1	1 (0.7–1.4)	0.883		
Don’t remember	16/30	53.3	1.6 (0.8–3.4)	0.219		
**STIs treated in the past 12 months**						
Yes	53/116	45.7	1 (ref)			
No	214/520	41.2	0.8 (0.6–1.2)	0.371		
Don’t know	5/7	71.4	3 (0.6–15.9)	0.204		
**HIV Status**						
Negative	116/368	31.5	1 (ref)			
Positive	157/276	56.9	**2.9 (2.1–4)**	**<0.001**	**4.1 (2.8–6.0**)	**<0.001**
**Interaction between HIV and ever had other STI (0 = negative, 1 = positive, 9 = unknown)**						
0 0	68/211				1 (ref)	
0 1	39/139				1.0 (0.6–1.7)	0.949
0 9	7/15				2.1 (0.7–6.4)	0.201
1 0	74/126				**4.6 (2.8–7.7)**	**<0.001**
1 1	74/133				**4.2 (2.6–7.1)**	**<0.001**
1 9	9/15				**4.1 (1.3–13.4)**	**0.020**

^i^Adjusted Odds Ratio for age marital status, level of education, occupation, history of pregnancy, age at first sex, history of STIs, STIs treated in the past 12 months, HIV status;

* negatively confounded by age, after adjustment for age this was no longer statistically significant and hence not retained in the final multivariable model

## Discussion

This is the first study assessing prevalence of and risk factors for hr-HPV infection in Swaziland, a country severely affected by the HIV epidemic. The present study shows a high prevalence of hr-HPV infection (46.2%), corresponding to 174,046 women (aged between 15 and 49 years old) with hr-HPV in Swaziland. Differences were observed regarding the HPV types, whereHPV31/33/35/52/58 group types had the highest frequency (26.7%) among the study population. Among the same population, high prevalence of HIV (42.7%) was found. The age-specific prevalence of hr-HPV significantly decreased with age, yet HIV prevalence increased with age and slightly declined in the 45–49 year age group. The hr-HPV/HIV co-infection was 24.4%, with a high peak in the 30s and decline in older groups. The prevalence of multiple hr-HPV infections was statistically significantly higher among HIV-positive women (27.7%) when compared with HIV-negative women. In our sensitivity analysis HPV16 and HPV31/33/35/52/58 types were significantly higher among HIV-positive women when compared to HIV-negative women. Multivariate analysis showed that HIV status was the strongest risk for hr-HPV infection (OR = 4.9). Also the risk of being infected with hr-HPV infection decreased with increasing age (OR = 0.92), and being married (OR = 0.4).

The key strength of our study was that primary data and new specimens were used to ascertain the study outcomes. Being the first hr-HPV study done in the country we were able to extrapolate population totals of the 15–49 age group from the 2007–2030 population projections aligned to the 2014 population estimates [20] to estimate the population burden of hr-HPV infection and weighted hr-HPV infection in this population. This was done because our study population was age-stratified and we needed to adjust sample characteristics to match up with the target population (15–49 years) that they were selected to represent. Furthermore, since we had seven age-groups in our sample with various numbers of participants, we assigned various weights (age and region) to the response, to improve our ability to extrapolate results which were as interpretable as possible. This was done to prevent unequal counts or skewed results. The study was done in Swaziland among normal women population. The women were asymptomatically or did not have any symptoms that could have led to selection bias. An important limitation is the lack of genotyping of all the HPV types including low-risk types circulating within the population. We can currently only generalize the results among the population of age 15–49 years in Swaziland. In addition, since our study subjects were recruited from hospitals, they may not be truly representative of the general population which could introduce selection bias. However, weighting was applied when reporting summary results for the whole study sample.

Our study shows a high overall prevalence of hr-HPV infection, with the highest among the younger age groups. These findings are consistent with previous studies reporting high HPV prevalence in the population of sub-Saharan Africa, Asia and Latin America [22–27]. A study done in South Africa demonstrated a hr-HPV prevalence of 44.9% among women without cytological abnormalities [27]. Studies conducted in Tanzania and Nigeria found high prevalence of HPV (20.1% and 14.7% respectively) [23, 25]. In China the prevalence of HPV infection was found to vary between regions from 13.8%–44.4% [28]. A higher prevalence of above 75% was observed in two studies conducted in Brazil [22, 26]. Furthermore, the prevalence found in this study is higher than that of the sub-Saharan Africa region (SSA) (24.0%) [10].

HPV prevalence is highest among women <25 years old with a rapid fall among women in their 30s and 40s [27]. As highlighted in two large meta-analyses of HPV prevalence studies in LICs [4, 24], the prevalence of HPV and hr-HPV in both groups was highest among young women with a steady decline until the age group of 45–49. Our study concurs with such findings where the age-specific prevalence was significantly high among the ages of 15–30 and declined with age. According to Winer and colleagues, the actual first peak of HPV infection might be before the age of 25 years, as women are usually infected after sexual initiation [29]. HPV16 and 18 types have been found to be the most prevalent high risk types in most studies [4, 26–28, 30–32], yet in the current study we were not able to establish that. However, we observed high frequency of hr-HPV31/33/35/52/58 group types among the study population. Therefore, we cannot draw conclusions as to which is the most common genotype within the groups detected because the Xpert-HPV test gives results from six separate channels and the lack of genotyping. However, the current study has provided background knowledge about the distribution of hr-HPV in the Swazi women population. Such information is crucial to guide the introduction of prophylactic vaccine, as the country is working towards achieving such a goal. Furthermore, HPV screening seems to promote earlier identification of women at high risk of CC.

In relation to screening, the main problem is that if the country decided to do, for example, HPV and treat this group they would be treating 40% of the 30–34 age group and 30% of the 35–44 age group, which is much more than what has been done in other places. In this context the country would definitely require a triage method or a reduction in the number of hr-HPV types included for primary screening. The high frequency of types rarely seen in cancer is particularly important.

In this study, HIV weighted prevalence was very high among the study population (42.7%) and there was a high prevalence in the 25–29, 30–34 and 35–39 age groups, (34.1%, 44.2%, 40.7% respectively). The increase in the prevalence of HIV as age increases has been reported in different surveys [33, 34]. The current study demonstrated the high HPV/HIV co-infection of 24.4%, with high hr-HPV prevalence across almost all age groups and more multiple hr-HPV infection in HIV-positive women than compared to HIV-negative women. These findings are consistent with previous studies reporting higher prevalence of HPV [9, 27, 30, 35] and higher frequency of infection with multiple HPV types among HIV infected women [30, 32, 36]. A number of risk factors for HPV infection among women have been demonstrated in previous studies, including having more than one partner, early sexual initiation, age and marital status. In the multivariate adjusted model, age was the variable with a strong inverse associated with HPV risk, which might be due to the fact that most young women get infected after sexual initiation [29] and exposure is reduced or immunity develops with increasing age. Based on our findings, the risk of hr-HPV infection decreased with increasing age. Therefore, older women were less likely to have hr-HPV infection. Such inverse association with age has been shown in numerous other studies [37–39]. After adjusting for all the possible socio-demographic and reproductive confounders, women living with partners were less likely to be infected with HPV infection than compared to single women. Our results are consistent with other studies, where women who were not married or living with a partner had a higher risk of HPV infection [40, 41].

Studies have demonstrated unemployment as significant driver for the increased STIs among women and it’s associated with significantly higher odds of STIs [42, 43]. However, our study has found contrasting findings where by significant association between employment status and hr-HPV infection was not established. This might be due to the fact that financial independent woman have the ability to provide financial and material resources for themselves even if they are in a relationship. Furthermore, such ability can give them power to make sound decisions about engaging in protected sex when compared to unemployed women. According to Muula, unemployment may leave women with limited alternatives where they may resort to engaging in high risk behaviour such as becoming sex workers or engaging in transactional sex in which they provide sex in exchange for money and material resources from a partner [44].

A vital clinical and public health consequence of our findings is that HIV was the strongest risk factor associated with hr-HPV infection. HIV-positive women are approximately 5 times more likely to be infected with hr-HPV infection. Additional, overall infection and specifically HPV16 and HPV31/33/35/52/58 were significantly more common among HIV-positive women as compare to HIV-negative women in our study. The strong association between HIV and HPV infection has been demonstrated in several previous studies [38, 45, 46]. The increased risk of HPV infection in HIV patients has been associated with impaired immune response to HPV [46]. On the other hand, HPV-DNA has been established to be at a level of 6–18 times more likely to persist among HIV-infected women than compared to HIV-negative women [47]. HIV affects the immune system, making individuals more susceptible to HPV infection, and less likely to clear their HPV infection [47, 48]. These findings are significantly important for polices with a focus on HIV infected women to prevent HPV infection, cervical lesions and CC.

We further assessed the interaction between HIV and history of STIs with hr-HPV. Our study found that the presence, absence or unknown of history of STI with HIV did not appear to modify the relationship with hr-HPV infection. To our knowledge no much studies that have assessed the interaction between HIV and history of STIs with hr-HPV. However, there is an evidence that STIs or having had STI 12months before increases the probability of HIV including HPV infection [49, 50].

Factors such as educational status, history of pregnancy, number of pregnancies, age at first sexual encounter, age at menarche, number of sexual partners, contraceptives use, history of STIs and history of STIs treated in these past 12 months were not associated with HPV infection in our study, although in other studies, the factors were strongly associated with among population at risk HPV infection [31, 40, 51, 52]. The explanation of such inconsistency may indicate the role of high risk partners.

In conclusion, the prevalence of hr-HPV is high among women of reproductive age in Swaziland. HIV prevalence among reproductive aged women remains high in the country. The study confirms the high HPV/HIV co-infection rate and the high correlation between hr-HPV and HIV infection observed in previous studies. In addition, HPV infection was significantly common among HIV-positive women as compared to HIV-negative women in all age groups. The prevalence of multiple hr-HPV infections was significantly high among HIV-positive women compared to HIV-negative women. Furthermore, the study has provided essential information about the HIV link with HPV infections which may explain the high incidence. This can contribute to policy development and planning of prevention strategies incorporating HPV infection prevention especially among the youth and HIV infected people. Furthermore, our findings support the suggestion of comprehensive screening approaches incorporating hr-HPV testing with the existing screening programme, must be considered with triage modalities other than cytology. Finally, our findings provide epidemiological knowledge about the distribution of hr-HPV. This is crucial to guide the future introduction of prophylactic vaccine in Swaziland.

## Supporting Information

S1 Fig Age-specific of hr-HPV prevalence among reproductive aged women by HIV status, Swaziland. (TIF)

S1 Table Prevalence and association of hr-HPV types with HIV status among the reproductive age women. (DOCX)

## Abbreviations

ASCUS: Atypical squamous cell of undermined significance
HPV: Human Papillomavirus
HSIL: high-grade squamous intraepithelial lesion
LSIL: Low-grade squamous intraepithelial lesion
Pap: Papanicolaou
VIA: Visual inspection with acetic acid
