# Supplementary figures and images for: Prevalence of and Associated Risk Factors for High Risk Human Papillomavirus among Sexually Active Women, Swaziland
Source: PLoS One. 2017 Jan 23;12(1):e0170189. doi: 10.1371/journal.pone.0170189 (PMC5256897; doi:10.1371/journal.pone.0170189)

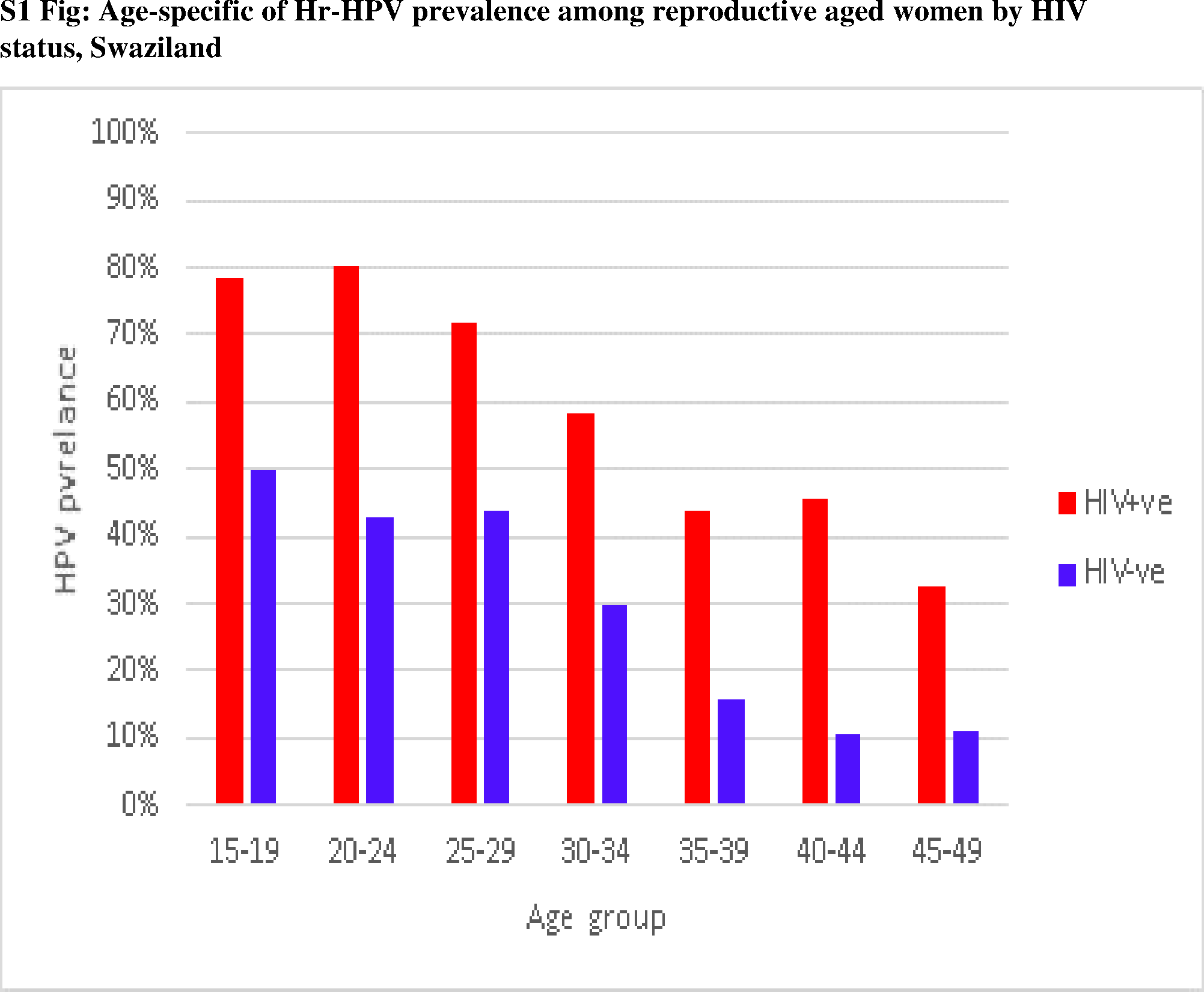

Supplement: S1 Fig — (TIF) [file pone.0170189.s001.tif]
