# Supplementary material for: Prevalence of and Associated Risk Factors for High Risk Human Papillomavirus among Sexually Active Women, Swaziland
Source: PLoS One. 2017 Jan 23;12(1):e0170189. doi: 10.1371/journal.pone.0170189 (PMC5256897; doi:10.1371/journal.pone.0170189)
Supplement: S1 Table — (DOCX) [file pone.0170189.s002.docx]

**S1 Table: Prevalence and association of hr-HPV types with HIV status among the reproductive age women.**

| **Hr-HPV Types** | | **HIV** | | **P-value** | **Univariate analysis**  **(OR, 95%CI)** | **P value** |
| --- | --- | --- | --- | --- | --- | --- |
|  |  | **Negative (%)** | **Positive (%)** |  |  |  |
| **HPV16** | **-ve** | 91.2 | 82.4 |  | 1 |  |
|  | **+ve** | **8.8** | **17.6** | **0.03** | **2.2 (1.1 – 4.5)** | **0.03** |
| ***Total*** |  | ***100*** | ***100*** |  |  |  |
| **HPV 18_45** | **-ve** | 89 | 82.1 |  | 1 |  |
|  | **+ve** | 11 | 17.9 | 0.077 | 1.7 (0.9 -3.3) | 0.002 |
| ***Total*** |  | ***100*** | ***100*** |  |  |  |
| **HPV 31/33/35/52/58** | **-ve** | 82.7 | 59.6 |  | 1 |  |
|  | **+ve** | **17.3** | **40.4** | **<0.001** | **3.2 (1.9 – 5.5)** | **<0.001** |
| ***Total*** |  | ***100*** | ***100*** |  |  |  |
| **HPV51/59** | **-ve** | 93.9 | 90.1 |  | 1 |  |
|  | **+ve** | 6.1 | 9.9 | 0.095 | 1.7 (0.9 – 3.2) | 0.097 |
| ***Total*** |  | ***100*** | ***100*** |  |  |  |
| **HPV39/68/56/66** | **-ve** | 90.7 | 86.4 |  | 1 |  |
|  | **+ve** | 9.3 | 13.6 | 0.150 | 1.5 (0.8 – 2.8) | 0.005 |
| ***Total*** |  | ***100*** | ***100*** |  |  |  |
